# Using autolyzed and dried *Saccharomyces cerevisiae* as various macromolecules and sucrose substitute: Developing novel gummy

**DOI:** 10.1016/j.fochx.2025.103045

**Published:** 2025-09-16

**Authors:** Tahra ElObeid, Burcu Tüzün, Ayşe Apaydın, Omer Said Toker, Caglar Doguer, Ibrahim Palabiyik, Muhammed Irfan Aksu, Nevzat Konar, İlyas Atalar

**Affiliations:** a Qatar University, Department of Nutrition Sciences, College of Health Sciences, QU Health, Doha, Qatar; b Eskisehir Osmangazi University, Agriculture Faculty, Food Engineering Department, Eskisehir, Turkey; c Yıldız Technical University, Faculty of Chemical and Metallurgical Engineering, Food Engineering Department, İstanbul, Turkiye; d Tekirdag Namik Kemal University, Health Sciences Faculty, Nutrition and Dietetics Department, Tekirdag, Turkey; e Tekirdag Namik Kemal University, Agriculture Faculty, Food Engineering Department, Tekirdag, Turkey; f Ataturk University Agriculture Faculty, Food Engineering Department, Erzurum, Turkey; g Ankara University Agriculture Faculty, Dairy Technology Department, Ankara, Turkey

**Keywords:** Autolyzed yeast cell, Bioavailability, Gelatine, Gummy, Confectionery

## Abstract

Autolyzed yeast cells contain a combination of various macromolecules such as mannan oligosaccharides, glucans, and manno protein, and they are noteworthy as a source of prebiotics and postbiotics. This study used spray drying to obtain *Saccharomyces cerevisiae* autolyzed yeast cells powder and replaced it with sucrose in a gummy formulation. The increase in the amount of autolyzed yeast also caused a significant increase in the total phenolic contents and antioxidant capacity. The optimum points for gummy ingredients were 26.27 % sucrose, 6.33 % autolyzed yeast cells, and 7 % gelatine concentrations, considering maximum springiness, bioactive properties, and minimum springiness, hardness, and ΔE changes during accelerated shelf-life storage. Optimum gummy formulation significantly reduced Caco-2 (55 %) and A549 (60 %) cell viability compared to the control group. It was concluded that yeast cells-added gummies could be a healthy substitute and could be commercialized due to their acceptable structural and sensorial properties.

## Highlights

• Autolyzed and dried yeast cell (ADYC) was used as sugar replacer in gummy formulations.
• The optimum formulation determined as were 26.27 % sucrose, 6.33 % ADYC, and 7 % gelatine
• Optimum gummy formulation significantly reduced Caco-2 (55 %) and A549 (60 %) cell viability.
• ADYC partially substitution sucrose and gelatin in gummy resulted with reduced water activity.

## 1 Introduction

In recent years, the re-regulation of sugar content in foodstuffs with high caloric value or acute glycemic response has been among the priorities of international health authorities (Soukoulis & Tzia, 2018). Also, confectionery consumers have demands and expectations for healthier and lower sugar content than traditional products, and innovative products in terms of appearance, texture, and flavor should be offered to the market in this product group (Periche et al., 2014). In recent years, the functional confectionery market has experienced significant growth. Estimated at USD 70.4 billion in 2024, the market is projected to reach USD 133.3 billion by 2034, with a compound annual growth rate (CAGR) of approximately 6.6 %. This growth is driven by increasing consumer awareness of health and wellness, integrating functional ingredients—such as vitamins, minerals, probiotics, and antioxidants—into confectionery products, and a growing preference for products that combine indulgence with nutritional value. Additionally, the demand for personalized dietary solutions, the rising interest in plant-based and allergen-free formulations, and advancements in food technologies further accelerate innovation and diversification within the market (Goradel, 2025). In this group of products, sugar can be fully or partially substituted. The main functions of sugar in foods are its effects on flavor and texture, Maillard reactions and various technological and quality-related properties. In addition, glucose syrup is a sweetener in gummy production, affects texture and microbial stability and stabilizes other ingredients such as sucrose or gelatin (Burey et al., 2009). However, substituting sucrose and glucose syrup in confectionery with healthier bulking agents results in a value-added product (Periche et al., 2014). Yeast cells and their wall materials are notable as a source of prebiotics because they contain a mixture of mannan oligosaccharides, glucans and manno protein (Ahiwe et al., 2021). The cell wall of *Saccharomyces* ssp. has a bilayer structure about 100–200 nm thickness, which externally surrounds the plasma membrane of yeast cells and constitutes 15–25 % of the cell's dry mass. Most of this dry mass consists of polysaccharides (80–90 %), especially mannans and glucans. Other cell wall structure components are proteins, lipids and inorganic phosphate (Nelson et al., 2006). *S.cerevisiae* cell mannans have been reported to have antioxidant, antibacterial and antimutagenic properties (Liu et al., 2018). Beta-glucan is classified as a bioactive compound due to its health-beneficial properties. The European Food Safety Authority (EFSA) recommends that the amount in the diet should be at least 3 g per day (Drozińska et al., 2019). Considering these health effects, it may have the potential for use both as a bulking agent and a bioactive sugar substitute to produce innovative health-promoting foods. The widespread consumption of confectionery is increasing due to its potential to be used as a delivery material for various bioactive compounds. The fact that *S. cerevisiae* cells also have a prebiotic effect is an essential innovation for gummy confectionery, and it can potentially provide a product that can meet consumers' expectations. Confectionery can be divided into two main categories, hard and soft confectionery, depending on the type of gelling agent and glucose syrup content in their formulation. Gelatin is a component that acts as a gelling agent, texture stabilizer and water binder. Soft candy can be defined as a biopolymer gel system formed in aqueous media. The addition of sugars to this system and the presence of these substances can cause changes in the structure of solid substances. One of the factors that has a significant impact on the textural properties of confectionery is moisture value. Because high moisture content can cause internal molecular mobility and recrystallization of polyol in confectionery, changes in the moisture level in confectionery can cause quality problems such as crystallization stickiness, development/acceleration of rancidity, chewiness differences, hardness, shaping problems, and textural defects (Burey et al., 2009). The effect of gelatin level in jelly-type confectionery formulation will be the first study regarding water binding and texture stabilizing effects during storage.

In this study, autolyzed yeast cell powders were produced using the spray drying technique. The substitution level of sucrose with dried *S. cerevisiae* cells and the optimum gelatin level were investigated using a mixture design that considered textural, color, physicochemical, and bioactive properties. In the final stage of the study, the aim was to verify the optimum formulations and to determine the stability of the color and textural properties of the samples to be prepared based on this formulation under accelerated shelf life conditions. Bioavailability analysis of the gummy samples obtained at optimum points was also carried out.

## 2 Material and methods

### 2.1 Materials

For the preparation of gummy samples, water, medium grain size sucrose (Konya Sugar, Konya, Turkey), 42-DE glucose syrup (Cargill, Bursa, Turkey), and 250 bloom g Type A gelatin (Gerede Jelatin, Bolu, Turkey), natural colorant (beetroot red, Sensient, Kocaeli, Turkey) and *Saccharomyces cerevisiae* NCYC-R-625 (Pakmaya, İzmit, Turkey) were used.

For preparation, autolyzed and dried *S. cerevisiae* NCYC-R-625, an instant yeast solution was prepared using deionized water (30.0 g/100 mL). The solution was then heated to a temperature of 80 °C. It was kept at this temperature for 2 h with gentle stirring. Following this, the drying process was carried out using the spray-dryer method. Spray drying was done in a lab-scale spray dryer (B290, Buchi, Fluwil, Switzerland). For the process, autolyzed and dried *S. cerevisiae* NCYC-R-625 solution was prepared (15.0 g/100 mL). Drying conditions were 6 bar gas pressure, 1.40 mL min-1 flow, and 65 mbar atomization pressure. The spray dryer's inlet and outlet temperature values were 180 °C and 100 °C, respectively. The dried samples were stored in tight, dark color packages at 20.0 ± 2.0 °C.

Autolyzed and dried *S. cerevisiae* cells were characterized and dry matter, crude protein, glucan-mannan and ash contents were determined as 94.5, 2.5 g/10 g, 40.0 ± 150. g/ 100 g, 33.0 ± 1.00 and 6.00 ± 0.75 g/100 g, respectively.

*S. cerevisiae* NCYC-R-625 was grown on a molasses-based medium and obtained after spray drying. The powder form of yeast cells has 94.5 ± 2.5 % dry matter content. The protein content is 40 %, the glucan-mannan content is 33 %, the ash content is 6 %, and the fat content is 1 %.

### 2.2 Gummy candy production

As control samples, gummy-type confectionery samples were prepared using water (17.4 g/100 g), sucrose (32.6 g/100 g), glucose syrup (41.7 g/100 g), 250 bloom gelatin solution (6.3 g/100 g), and natural colorant (beetroot red, 300 mg/100 g). To prepare the control samples and the samples in which dried yeast cells were used as sucrose substitutes, water, sucrose, and glucose syrup were heated in a thermal blender (Thermomix, Vorwerk, Wuppertal, Germany) with stirring (300 rpm) until reaching a boiling point (10 min). The sample was then cooled to 60 °C, and °Brix was measured with a hand-type digital refractometer (Abba, Atago, Tokyo, Japan). The previously prepared 250-bloom gelatin: water solution (1:2) was mixed by adding the solution, followed by adding colorant and stirring at 3000 rpm at 60 °C for another 5 min. At the end of the time, the syrup was transferred to silicone molds and kept at 4 °C for 24 h. To ensure standardization, particularly in texture analyses, samples were deposited into silicone molds suitable for producing cylindrical gummy specimens with a diameter of 28 mm and a height of 20 mm, considering the probe diameter used in the analysis method. The samples taken from the molds were coated with starch to prevent sticking to each other and placed in polyethylene bags.

### 2.3 Textural analysis

Confectionery samples were prepared with a diameter of 28 mm and a height of 20 mm and then subjected to Texture Profile Analysis (TPA) (TA-XT, Stable Microsystems, New Castle, USA). The instrument includes a 5 kg load cell and a 35 mm diameter cylindrical probe. Compression of 50 % was performed twice at intervals of 15 s. The test speed was one mm/s. Hardness (N), springiness, cohesiveness, and gumminess (N) values were determined using the force-time curves.

### 2.4 Physicochemical and color analysis

The color properties of confectionery samples were examined using the CIE-Lab method (L*, a*, b*, C*, and h°) with a colorimeter (CR-4000, Konica Minolta, Japan). The L* (luminance), a* (± red-green), and b* (± yellow-blue) values of the samples were measured, and the chroma (C*) and hue angle (h°) values of the samples were determined using these values. Analyses were carried out in five replicates. The pH of the gummy samples was measured using a pH meter (HI2211, Hanna, Woonsocket, USA) after the samples were cut into thin slices and mixed with water. Water activity was measured with a water activity meter (4TE, Aqualab, Pullman, USA) at 25 °C. 2.5 g of gummy samples were weighed into weighing cups and dried in an oven at 105 °C for 24 h for dry matter analysis.

### 2.5 Bioactive characterization

#### 2.5.1 Extraction

The methods of Muzzaffar et al. (2016) were adapted to obtain gummy extracts to perform the total phenolic acid amounts and DPPH free radical scavenging activity analyses. Two grams of gummy samples were weighed and taken into 50 mL centrifuge tubes. The samples were shredded in a homogenizer at 500 rpm for 1 min by adding a 20 mL (50:50 methanol: distilled water) solution. The samples were shaken in a water bath at 240 rpm for 1 h, centrifuged at 4000 rpm for 10 min in a centrifuge, and filtered through 150 μm filter paper into tubes. The resulting confectionery extracts were stored in the refrigerator at −18 °C.

#### 2.5.2 Total phenolic contents

First, 0.5 mL of gummy extracts was mixed with 2.5 mL of Folin–Ciocalteu's phenol reagent (10 %). Then, 2 mL of Na_2_CO_3_ aqueous solution (%7.5 *w*/*v*) was added to the mixture. After 30 min of reaction at room temperature, the mixture was centrifuged (3000 rpm for 5 min), filtered through a 0.22 μm pore size filter, and the absorbance was read with a spectrophotometer (Jasco UV/Vis Spectrophotometer, Japan) at 765 nm (Singleton et al., 1965). The results were calculated using a standard gallic acid (GA) solution curve. The total phenolic content was expressed as milligrams of gallic acid equivalents (GAE) per kilogram (mg GAE/kg). All measurements were performed in triplicate.

#### 2.5.3 Antioxidant capacity

4.9 mL of a 0.1 mM DPPH solution and 0.1 mL of the gummy extract were mixed and then incubated for 30 min. The absorbance was read with a spectrophotometer (Jasco UV/Vis Spectrophotometer, Japan) at 517 nm. The results are expressed as inhibition %.

(1) $$\%\mathrm{Inhibition}=\left[\left(A_{C}-A_{T}\right)/A_{C}\right]\;x\;100$$

Ac; Control absorbance value, At; sample absorbance value.

#### 2.5.4 *In vitro* bioactivity analysis

Human colon (Caco-2) and lung (A549) carcinoma cell lines sourced from the American Type Culture Collection (ATCC) were cultured in minimum essential medium (MEM) with Earle's salt (Sigma M4655) supplemented with fetal bovine serum (15 % (*v*/v); Capricorn Scientific; FBS-11 A), sodium pyruvate (1 % v/v; Sigma S8636), nonessential amino acids (1 % v/v; Sigman 7145), and antibiotic mixture (1 % v/v) containing amphotericin B, streptomycin, and penicillin (Sigma A5955), to provide an optimal environment conducive to cell growth and proliferation *in vitro*. Cells were maintained under standard conditions in a humidified incubator at 37 °C with 5 % CO_2_. To investigate the inhibitory effects of gummy and yeast cell samples on cancer cell viability, the 3-(4,5-Dimethylthiazol-2-yl)-2,5-diphenyltetrazolium bromide (MTT) assay was performed following the previously established protocol. An estimated count of 1 × 105 cells per well, determined using a Thoma chamber, was plated into each 96-well culture plate, where they proliferated for 24 h. The cells were treated with 80 μL of gummy and yeast cell samples and incubated for 24 or 48 h. Before all experimental treatments, all samples were filtered using a syringe filter with a 0.45 μm pore size. The MTT assay was performed following treatment procedures. This involved the addition of MTT solution to each well, followed by further incubation to allow viable cells to convert the yellow MTT into purple formazan crystals. DMSO solubilized the resulting formazan crystals, and each well's optical density (OD) was measured at a wavelength of 570 nm using a microplate reader (Shimadzu: UV-2600 Spectrophotometer). To calculate the percentage of cell viability, the OD value of the treated sample was subtracted from the OD value of the blank, and the result was divided by the difference between the OD value of the control and the OD value of the blank. This value was then multiplied by 100.

### 2.6 FTIR analysis

Spectral measurements were performed within the range of 4000–500 cm^−1^. All measurements were performed in triplicate with a diamond triple-bounce ATR accessory (Bruker Alpha, Germany).

### 2.7 Sensory analysis

The sensory characteristics (appearance, color, aroma, sweetness, texture, firmness, chewiness, and odor) of the confectionery samples will be determined using a 9-point hedonic scale (1 = dislike, 9 = like very much) and semi-trained panelists who are 40 undergraduate students from the Food Engineering Department at Eskişehir Osmangazi University. (Sanz et al., 2009).

### 2.8 Accelerated shelf life study

The gummy samples were placed in the climate chamber without packaging the day after their preparation. They kept at 75 % RH at 25 °C based on the accelerated shelf life test conditions for confectionery samples. At 7-day intervals, L* (brightness), a* (± red-green), and b* (± yellow-blue) values of the samples were measured with a colorimeter (CR-400, Konica Minolta). In addition, the samples were subjected to Texture Profile Analysis (TPA) for the same periods.

### 2.9 Statistical analysis

The experimental design was planned using the D-Optimal design to determine the optimum processing conditions for gummy production. The independent parameters were selected as sucrose content, dried autolyzed cell content, and gelatine content. The effects of independent variables on textural, color, bioactive and physicochemical properties were determined. After determining the optimum points for targeted response values, the model validation of gummy samples was conducted to determine accuracy and precision. Accelerated shelf life and bioavailability analyses were performed for the jellies produced at the optimum point and control samples.

## 3 Results and discussion

### 3.1 Texture properties

Texture is one of the main quality parameters in confectionery products. There is a strong relationship between textural properties and product categorization. For example, texture properties form the basis of categories such as hard and soft candies and chewy candies. In addition, there is a remarkable correlation between textural properties and sensory properties, sugar crystallization, moisture content, and water activity parameters (Gunes et al., 2022). The effects of the independent variables on the texture characteristics of gummy samples were examined with the determination of hardness (1096.5–2040.1 g), resilience (49.2–88.9 %), cohesion (94.3–101.7), springiness (26.1–48.2 %), and chewiness (37,845–87,611 g) parameters (Table 1). Additionally, significant models were identified regarding the effect of independent variables on all these parameters with higher R^2^ values of 0.7897 and 0.9766 (*P* < 0.05). TPA values of gummy samples are generally compatible with previous studies that studied gellies prepared under different compositions and conditions (Gok et al., 2020; Gümüş et al., 2023; Kahraman et al., 2023; Kurt et al., 2022; Teixeira-Lemos et al., 2021). In the literature, chewiness values of gummy samples vary within wide ranges. Chewiness results are consistent with the study in which vitamin C release was examined in samples prepared using different amounts of gelatin (3.0–12.0 %) (Zhou et al., 2022). Another study examining the usage possibilities of different hydrocolloids contains similar results (de Avelar & Efraim, 2020). However, the obtained results should be discussed by considering other texture parameters. It can be noted that with this approach, negative results were obtained for chewiness. Because an increase in this value means an increase in the spent power on chewing during consumption (Burey et al., 2009). Therefore, some consumers may consider this a negative feature. The possible reason for this result can be associated with the product formulation. The chewiness value of the sample containing only sucrose and gelatin, which can be defined as the control sample, is also significantly higher than in various previous studies. In previous studies, various organic acids, especially citric acid, were used in the composition of the gummy, but they were not included in the composition in this study. This can be mentioned as one of the factors that could impact Chewiness values. In addition, it was determined that all independent variables and sucrose x autolyzed yeast cell and gelatin x autolyzed yeast cell interactions (Table 2) had a significant effect on the chewiness value (*P* < 0.05). Hardness is the term used to describe the gel structure's resistance to pressure. It is employed to assess how food feels in the mouth. The main effect of gelatin content was important in the hardness of gummy samples (*p* < 0.0001). Also, in previous studies, an increase in gelatin concentration results in a concurrent increase in gel hardness (Aidat et al., 2023; Noh et al., 2023). The interaction between gelatin, sucrose, and autolyzed yeast cell content significantly influenced gummy hardness (Table 2). The lowest hardness values were observed in formulations containing 5 % gelatin and 2.7 % autolyzed yeast cells, indicating that both network-forming capacity and water-binding properties are critical determinants of texture. Sucrose plays a dual role by acting as a plasticizer and providing binding sites for gelatin network formation, resulting in a denser and mechanically stronger structure (Dalabasmaz et al., 2023). When sucrose was partially replaced with autolyzed yeast cells, hardness decreased slightly; however, these changes were within a tolerable range for product acceptability. This suggests that textural properties can be compensated by adjusting gelatin concentration without compromising product integrity, a strategy also reported in sugar-reduced gummies formulated with alternative polysaccharides (Aidat et al., 2023; Noh et al., 2023).

**Table 1:** Texture profile analysis of gummy samples.

Run	Sucrose (g/100 g)	Dried Yeast Cell (g/100 g)	Gelatin (g/100 g)	Hardness (g)	Resilience (%)	Cohesion (%)	Springiness (%)	Gumminess (g)	Chewiness (g)
1	32.60	0.00	7.00	2040.1 ± 58.4	88.1 ± 0.40	99.6 ± 0.2	35.8 ± 0.66	181,814 ± 4736	65,141 ± 1593
2	24.50	8.10	8.00	1897.4 ± 59.8	61.0 ± 1.46	94.3 ± 0.41	38.1 ± 3.49	187,111 ± 6432	70,976 ± 4078
3	27.20	5.40	8.00	1559.6 ± 15.9	69.6 ± 1.95	96.5 ± 0.41	47.1 ± 1.24	139,377 ± 18,325	65,922 ± 10,368
4	32.60	0.00	6.50	1715.1 ± 5.98	88.9 ± 0.54	99.7 ± 1.50	48.2 ± 5.45	183,960 ± 20,804	87,611 ± 3306
5	24.50	8.10	6.00	1571.0 ± 10.2	59.0 ± 1.55	97.3 ± 0.63	40.8 ± 3.80	182,839 ± 10,198	74,253 ± 3412
6	24.50	8.10	6.00	1517.0 ± 67.8	58.5 ± 1.47	95.6 ± 0.34	41.9 ± 4.48	144,974 ± 6619	60,479 ± 5088
7	24.50	8.10	6.00	1497.3 ± 41.6	60.7 ± 0.71	96.1 ± 0.81	36.2 ± 0.79	143,804 ± 3528	52,029 ± 929.7
8	24.50	8.10	6.00	1500.0 ± 189.9	61.5 ± 1.40	96.2 ± 0.95	33.1 ± 3.47	144,482 ± 19,799	47,090 ± 1882
9	32.60	0.00	7.00	1635.3 ± 22.1	86.9 ± 0.92	100.5 ± 1.05	33.7 ± 0.31	157,531 ± 9028	53,037 ± 2592
10	24.50	8.10	5.00	1447.1 ± 41.3	51.5 ± 2.67	97.5 ± 0.37	28.0 ± 2.92	141,083 ± 4554	39,373 ± 2842
11	24.50	8.10	6.00	1556.1 ± 55.3	60.1 ± 0.65	96.6 ± 1.42	34.3 ± 1.69	150,390 ± 7553	51,425 ± 45.2
12	24.50	8.10	6.00	1488.8 ± 152.7	56.8 ± 1.04	97.2 ± 0.28	28.4 ± 2.10	144,742 ± 14,829	40,885 ± 2259
13	29.90	2.70	5.00	1096.5 ± 2.51	71.1 ± 0.99	99.0 ± 0.84	32.1 ± 2.84	119,088 ± 15,080	37,845 ± 1188
14	24.50	8.10	6.50	1521.3 ± 96.7	62.7 ± 1.09	97.2 ± 0.33	40.2 ± 2.78	147,924 ± 9672	59,174 ± 147.9
15	32.60	0.00	5.00	1699.0 ± 33.5	77.2 ± 2.77	101.7 ± 1.25	26.1 ± 0.19	172,743 ± 4161	45,057 ± 1058
16	28.55	4.05	6.50	1888.1 ± 0.12	64.2 ± 2.37	97.8 ± 0.25	29.9 ± 2.18	195,446 ± 14,926	58,102 ± 2451
17	29.90	2.70	8.00	1761.2 ± 23.6	69.3 ± 2.01	97.8 ± 0.52	35.0 ± 7.69	166,002 ± 10,024	57,426 ± 9350
18	24.50	8.10	5.00	1751.1 ± 48.7	49.2 ± 1.98	97.2 ± 0.47	27.9 ± 3.28	161,405 ± 13,802	44,505 ± 1242
19	26.53	6.08	5.75	1676.5 ± 13.5	56.9 ± 2.10	96.5 ± 1.34	26.5 ± 1.89	168,788 ± 12,304	44,772 ± 5160

**Table 2:** ANOVA table showing the effects of linear, quadratic and interaction terms on each response individually.

	Hardness (g)		Resilience (%)		Cohesion (%)		Springiness (%)		Gumminess (g)		Chewiness (g)	
	*SS*	*P value*	*SS*	*P value*	*SS*	*P value*	*SS*	*P value*	*SS*	*P value*	*SS*	*P value*
*M*	6.14E ± 05	0.001	2537	< 0.0001	43.0	< 0.0001	1902	0.003	5.71E ± 09	0.019	7.06E ± 09	0.002
*X*_*1*_	11,141	0.360	36.2	0.027	0.869	0.312	217.3	0.030	3.43E ± 07	0.710	7.82E ± 08	0.045
*X*_*2*_	11,131	0.360	36.1	0.027	0.871	0.312	217.3	0.030	3.42E ± 07	0.710	7.82E ± 08	0.045
*X*_*3*_	3.51E ± 05	0.000	115.7	0.001	1.89	0.143	1007	0.000	3.06E ± 09	0.004	4.15E ± 09	0.000
*X*_*1*_*X*_*2*_	95,338	0.017	47.4	0.014	nd	nd	140.8	0.069	6.57E ± 08	0.121	7.57E ± 08	0.048
*X*_*1*_*X*_*3*_	3.50E ± 05	0.000	65.3	0.006	nd	nd	458.4	0.004	3.09E ± 09	0.004	nd	nd
*X*_*2*_*X*_*3*_	3.50E ± 05	0.000	65.3	0.006	nd	nd	nd	nd	3.09E ± 09	0.004	9.11E ± 08	0.033
*X*_*1*_^*2*^	nd	nd	nd	nd	nd	nd	140.8	0.069	nd	nd	nd	nd
*X*_*2*_^*2*^	nd	nd	47.4	0.014	nd	nd	140.8	0.069	nd	nd	7.56E ± 08	0.048
*X*_*3*_^*2*^	nd	nd	nd	nd	nd	nd	126.3	0.083	nd	nd	nd	nd
*Lf*	95,388.24	0.2395	39.88	0.171	9.37	0.143	213.6	0.1476	1.09E ± 09	0.709	1.18E ± 09	0.269
*R*^*2*^		0.806	*R*^*2*^	0.977	*R*^*2*^	0.783	*R*^*2*^	0.8485	*R*^*2*^	0.669	*R*^*2*^	0.790
*Adj- R*^*2*^		0.710	*Adj- R*^*2*^	0.962	*Adj- R*^*2*^	0.740	*Adj- R*^*2*^	0.7272	*Adj- R*^*2*^	0.503	*Adj- R*^*2*^	0.685

M; model, nd; not determined, Adj-R^2^; Adjusted R^2^, Lf; Lack of fit. *p* < 0.05.

The effect of autolyzed yeast on hardness is likely linked to its composition, particularly hydrophilic proteins, polypeptides, and β-glucans, which modify the moisture distribution and interaction within the gel matrix. Similar effects of high molecular weight biopolymers on confectionery texture have been observed in hydrocolloid-enriched systems (Burey et al., 2009). In our study, resilience and springiness—two parameters strongly associated with gel elasticity and consumer-perceived chewiness (Zhang et al., 2023)—were also significantly affected by all independent variables and their interactions (*P* < 0.05). This demonstrates that the structural role of autolyzed yeast extends beyond simple sucrose replacement, influencing network flexibility and water retention, which are key attributes for final texture perception.

The cohesiveness value is used to evaluate the extent to which the product withstands a deformation applied a second time after the first deformation, compared to the first deformation (using the area under the force-time diagram). The soft confectionery is easy to chew and swallow at low stickiness values. Cohesiveness has been accepted as an important factor among the acceptance criteria for many years (Dalabasmaz et al., 2023). However, in our study, a much wider range of changes was determined on chewiness, and no strong correlation could be detected between the two parameters. Overall, the incorporation of spray-dried autolyzed yeast cells induced measurable changes in the textural properties of gummy formulations. These changes can be attributed primarily to the reduction in sucrose, which serves as a structural binder for gelatin networks, and to the high moisture affinity of yeast-derived biopolymers such as β-glucans and mannoproteins. These components likely altered the water distribution and hydrogen bonding dynamics within the gel matrix, resulting in modified hardness, springiness, and resilience profiles. Similar texture modifications have been reported in sugar-reduced confectionery systems enriched with hydrocolloids or proteinaceous bulking agents, where the altered polymeric interactions necessitated formulation adjustments to restore network elasticity (Aidat et al., 2023; Burey et al., 2009). Notably, the observed increase in chewiness, often perceived negatively by consumers, can be effectively mitigated by optimizing gelatin concentration, supporting the feasibility of yeast-based formulations without compromising consumer-acceptable textural performance.

### 3.2 Physicochemical properties of gummy samples

In confectionery products, the amount of moisture in the final product affects the main quality characteristics, texture, sensory properties, and stability (Ergun et al., 2010; Gok et al., 2020). Additionally, the amount of moisture and dry matter is considered in product categorization (Gunes et al., 2022). The critical limit for gummy was determined as 76.0 g/100 g. In our study, the total dry matter amounts of the samples varied between 78.6 and 84.1 g/100 g (Table 3). Therefore, it was determined that all samples had a dry matter content higher than the critical amount for gummy, and they generally complied with the dry matter amounts specified for these products (78.0–92.0 g/100 g) (Hartel et al., 2018).

**Table 3:** Physicochemical and bioactive properties of gummy samples.

Run	Sucrose (g/100 g)	Dried Yeast Cell (g/100 g)	Gelatin (g/100 g)	Brix (°Bx)	Dry Matter Content (g/100 g)	Water Activity	pH	L*	a*	b*	C*	Hue Angle	Total Phenolics Content (mg GAE/kg)	Antioxidant Capacity Inhibition %
1	32.60	0.00	7.00	86.8 ± 0.01	80.57 ± 0.1	0.74 ± 0.00	5.72 ± 0.01	75.9 ± 1.13	15.4 ± 0.20	1.73 ± 0.15	15.5 ± 0.20	6.43 ± 0.52	26.6 ± 1.23	0.54 + 0.18
2	24.50	8.10	8.00	86.0 ± 0.04	79.68 ± 0.44	0.76 ± 0.01	5.55 ± 0.02	83.5 ± 0.70	5.59 ± 0.40	10.1 ± 0.85	11.5 ± 0.94	60.9 ± 0.36	45.3 ± 0.14	17.3 + 0.19
3	27.20	5.40	8.00	86.5 ± 0.03	79.43 ± 0.13	0.76 ± 0.01	5.58 ± 0.01	84.6 ± 0.16	6.57 ± 0.05	8.93 ± 0.16	11.1 ± 0.15	53.6 ± 0.30	39.9 ± 0.43	12.1 + 0.05
4	32.60	0.00	6.50	86.5 ± 0.02	82.2 ± 0.21	0.76 ± 0.01	5.80 ± 0.02	64.9 ± 1.25	14.4 ± 0.53	2.33 ± 0.16	14.5 ± 0.54	9.18 ± 0.31	28.9 ± 0.38	0.30 + 0.08
5	24.50	8.10	6.00	81.7 ± 0.01	78.62 ± 0.05	0.79 ± 0.00	5.63 ± 0.02	74.8 ± 0.65	3.95 ± 0.26	11.4 ± 0.48	12.1 ± 0.45	70.9 ± 1.38	42.6 ± 0.14	14.7 + 0.38
6	24.50	8.10	6.00	82.9 ± 0.02	81.04 ± 0.33	0.75 ± 0.02	5.60 ± 0.02	77.5 ± 0.13	5.73 ± 0.08	10.3 ± 0.26	11.8 ± 0.22	61.0 ± 0.77	47.6 ± 0.52	14.5 + 0.12
7	24.50	8.10	6.00	82.2 ± 0.01	79.82 ± 0.33	0.76 ± 0.01	5.67 ± 0.02	81.3 ± 0.13	4.06 ± 0.09	13.4 ± 0.20	14.0 ± 0.21	73.1 ± 0.27	47.9 ± 0.42	12.0 + 0.07
8	24.50	8.10	6.00	86.1 ± 0.03	80.62 ± 0.28	0.76 ± 0.02	5.65 ± 0.01	81.1 ± 0.41	5.59 ± 0.29	11.4 ± 0.39	12.7 ± 0.44	64.0 ± 0.91	45.7 ± 0.36	14.1 + 0.20
9	32.60	0.00	7.00	87.0 ± 0.01	83.81 ± 0.23	0.74 ± 0.01	5.56 ± 0.02	81.1 ± 1.60	13.8 ± 0.82	2.12 ± 0.23	10.0 ± 0.78	12.4 ± 1.92	24.8 ± 0.15	2.96 + 0.10
10	24.50	8.10	5.00	84.3 ± 0.02	81.9 ± 0.03	0.72 ± 0.02	5.47 ± 0.02	77.4 ± 0.85	7.74 ± 0.13	11.2 ± 0.36	13.6 ± 0.36	55.4 ± 0.54	29.8 ± 0.37	12.3 + 0.24
11	24.50	8.10	6.00	85.5 ± 0.01	81.31 ± 0.16	0.75 ± 0.01	5.56 ± 0.00	79.2 ± 0.31	6.08 ± 0.31	13.2 ± 0.36	14.6 ± 0.24	65.3 ± 1.59	41.1 ± 0.49	14.6 + 0.50
12	24.50	8.10	6.00	84.9 ± 0.04	82.02 ± 0.43	0.76 ± 0.02	5.60 ± 0.01	79.4 ± 0.21	7.54 ± 0.23	12.1 ± 0.20	14.2 ± 0.16	58.0 ± 1.05	46.6 ± 0.63	13.9 + 0.08
13	29.90	2.70	5.00	79.9 ± 0.03	84.15 ± 0.14	0.78 ± 0.02	5.70 ± 0.01	61.5 ± 0.82	7.56 ± 0.39	7.07 ± 0.22	10.4 ± 0.4	43.1 ± 1.12	47.9 ± 0.58	6.97 + 0.19
14	24.50	8.10	6.50	85.4 ± 0.01	80.45 ± 0.49	0.79 ± 0.00	5.66 ± 0.00	79.1 ± 0.45	7.99 ± 0.51	11.6 ± 0.47	14.1 ± 0.53	55.4 ± 1.76	35.7 ± 0.74	17.4 + 0.14
15	32.60	0.00	5.00	80.0 ± 0.01	83.59 ± 0.18	0.68 ± 0.01	5.47 ± 0.02	52.9 ± 9.39	11.4 ± 2.00	1.63 ± 0.65	11.6 ± 2.07	7.78 ± 1.85	47.3 ± 0.62	1.74 + 0.39
16	28.55	4.05	6.50	85.1 ± 0.01	83.04 ± 0.07	0.69 ± 0.0	5.75 ± 0.01	79.2 ± 0.72	9.82 ± 0.58	9.32 ± 0.13	13.5 ± 0.50	43.5 ± 1.30	20.7 ± 0.25	13.0 + 0.05
17	29.90	2.70	8.00	87.5 ± 0.01	83.79 ± 0.08	0.68 ± 0.01	5.50 ± 0.02	86.8 ± 0.73	6.29 ± 0.35	6.37 ± 0.31	8.95 ± 0.46	45.4 ± 0.31	38.2 ± 0.18	7.00 + 0.02
18	24.50	8.10	5.00	83.0 ± 0.02	82.21 ± 0.03	0.71 ± 0.00	5.59 ± 0.01	76.5 ± 1.84	8.08 ± 0.33	12.3 ± 0.96	14.7 ± 0.95	56.6 ± 1.43	29.5 ± 0.36	14.68 + 0.32
19	26.53	6.08	5.75	85.1 ± 0.01	82.09 ± 0.09	0.69 ± 0.00	5.59 ± 0.01	77.7 ± 1.81	8.27 ± 0.27	11.9 ± 0.93	14.5 ± 0.86	55.2 ± 1.80	43.9 ± 0.68	12.61 + 0.25

The replacement of sucrose with autolyzed yeast cells and the adjustment of gelatin concentration significantly influenced the dry matter content of the gummies (*P* < 0.05). Given that processing and post-processing conditions were kept constant, these compositional variables likely altered moisture diffusion dynamics during starch coating and gelation. Specifically, yeast-derived β-glucans, present predominantly as water-insoluble, high-molecular-weight fractions (Liu et al., 2021), are known to interact strongly with water and modify its mobility within food matrices. This property may have contributed to differences in water retention within the gelatin network, resulting in a distinct moisture loss pattern during the gelation phase. Similar findings have been reported in hydrocolloid- and fiber-enriched confectionery systems, where increased water-binding capacity altered drying kinetics and final moisture distribution (Ergun et al., 2010; Gok et al., 2020). These results suggest that autolyzed yeast cells act not only as a sugar substitute but also as a structuring agent influencing water partitioning and matrix stability, thereby requiring tailored formulation strategies to maintain desired dry matter levels.

Additionally, the shift in sucrose concentration affects the gelatin networks and the water held in this network structure (Gok et al., 2020). Therefore, it can be expected that the amount of autolyzed cells used will affect the moisture content of the final product. Accordingly, changes in other quality characteristics will be observed. However, it should be considered that autolyzed yeast cells are a complex material. It has been determined that the protein fractions in the composition of this material have strong hydrophilic interactions with water. It has also been found that autolyzation reduces the fat-binding capacity of yeast cells (Bertolo et al., 2019). Therefore, it is possible to use different fractions of this material, considering their effects on the final product's moisture content. Water activity is critical for gummy samples' microbiological and physical stability. The relationship of this feature with Tg and its consequent effects on crystallization behavior and tendencies should be considered in reformulation studies (Ergun et al., 2010). Although the water activity for gummy generally varies between 0.50 and 0.75 (Hartel et al., 2018), samples with different water activity values are obtained depending on the component, especially in sucrose replacement studies (Gok et al., 2020; Gunes et al., 2022). The study obtained samples with relatively higher water activity (0.68–0.79). Additionally, a significant model was identified for the effects of the independent variables on water activity values (*P* < 0.05) (Table 4). Especially the amount of sucrose and the use of autolyzed yeast are effective on this feature, and the interactions of these components with each other and gelatin were found to have significant effects on water activity (P < 0.05). The increase in the amount of autolyzed yeast cells and the decrease in sugar concentration resulted in a gummy with higher water activity. Using more gelatin with fewer yeast cells resulted in lower water activity. In general, the components in the yeast composition have a higher molecular weight than sucrose (Pereira et al., 2021). Using low-molecular components in confectionery composition results in products with lower water activity (Ergun et al., 2010).

**Table 4:** ANOVA table showing the effects of linear. Quadratic and interaction terms on each response individually.

	Total Phenolic Content		Antioxidant Activity		L*		a*		b*		C*		Hue Angle		Water Activity	
	*SS*	*P value*	*SS*	*P value*	*SS*	*P value*	*SS*	*P value*	*SS*	*P value*	*SS*	*P value*	*SS*	*P value*	*SS*	*P value*
*M*	3005	< 0.0001	528.5	<0.0001	910.5	<0.0001	174.4	< 0.0001	303.5	< 0.0001	42.6	0.022	9177	< 0.0001	0.018	0.003
*X*_*1*_	0.396	0.778	14.6	0.025	3.11	0.534	13.0	0.021	0.074	0.770	2.25	0.296	18.8	0.476	0.002	0.053
*X*_*2*_	0.388	0.781	14.6	0.025	3.12	0.771	12.9	0.021	0.075	0.768	2.24	0.297	18.7	0.477	0.002	0.053
*X*_*3*_	9.84	0.173	5.79	0.138	755.8	< 0.0001	0.550	0.598	2.79	0.087	0.138	0.792	8.93	0.621	0.000	0.421
*X*_*1*_*X*_*2*_	nd	nd	nd	nd	nd	nd	nd	nd	10.9	0.003	nd	nd	nd	nd	0.013	0.000
*X*_*1*_*X*_*3*_	nd	nd	nd	nd	229.3	< 0.0001	18.6	0.008	nd	nd	8.94	0.049	508.9	0.002	0.010	0.001
*X*_*2*_*X*_*3*_	nd	nd	nd	nd	nd	nd	18.6	0.008	nd	nd	8.94	0.049	508.9	0.002	0.010	0.001
*X*_*3*_^*2*^	nd	nd	nd	nd	nd	nd	nd	nd	nd	nd	15.2	0.014	nd	nd	nd	nd
*Lf*	38.3	0.781	27.5	0.155	77.1	0.221	16.0	0.287	5.03	0.862	17.2	0.211	322.7	0.337	0.004	0.076
*R*^*2*^		0.975	*R*^*2*^	0.937	*R*^*2*^	0.913	*R*^*2*^	0.877	*R*^*2*^	0.961	*R*^*2*^	0.633	*R*^*2*^	0.949	*R*^*2*^	0.773
*Adj- R*^*2*^		0.970	*Adj- R*^*2*^	0.925	*Adj- R*^*2*^	0.889	*Adj- R*^*2*^	0.829	*Adj- R*^*2*^	0.950	*Adj- R*^*2*^	0.464	*Adj- R*^*2*^	0.931	*Adj- R*^*2*^	0.660

M; model, nd; not determined, Adj-R^2^; Adjusted R^2^, Lf; Lack of fit. *p* < 0.0.

Changes in water activity are critical in determining sucrose crystallization behavior, which must be avoided in gummy systems due to its adverse effects on textural integrity and consumer perception (Gunes et al., 2022). In sucrose-reduced formulations, the altered osmotic balance and increased proportion of high-molecular-weight components can shift water distribution, influencing crystallization kinetics. Water activity gradients also serve as the primary driving force for moisture migration between the confection and its surrounding environment. When these gradients are pronounced, moisture migration accelerates, leading to structural defects such as surface hardening and the development of a dehydrated “skin” layer (Ergun et al., 2010). The presence of yeast-derived polysaccharides and proteins may further modify diffusion resistance, altering moisture migration rates compared to conventional sucrose-gelatin matrices. These findings highlight the necessity of carefully balancing water activity when incorporating autolyzed yeast to ensure textural stability and prevent crystallization-induced quality deterioration during storage. According to the results, it may be necessary to increase the gelatin concentration to prevent this risk when using high amounts of autolyzed yeast. However, in this case, products with high hardness are obtained. Therefore, glycerol, sorbitol and/or invert sugar can be used in these products as an alternative approach. In addition, accelerated shelf life studies determined a generally lower rate of hardness change (Supplemantary File 1).

The pH values of gummy samples varied within a narrow range (5.47–5.80). In general, pH values are controlled in confectionery products for various reasons, including modifying the aroma perception, preventing gelation, crystallization, and gel stabilization (Efe & Dawson, 2022). Additionally, adding various organic acids and components with a high affinity for water, such as glycerol, can reduce water activity while maintaining the textural quality of the product. For example, commonly used fruit acids such as citric and tartaric acids are hygroscopic. The study found higher pH values for gummy candies than in previous studies. pH values determined for gummy samples in various studies are in the range of 3.0–4.0 (Çoban et al., 2021; Sumonsiri et al., 2021; Ventura et al., 2013).

However, no acidity-regulating component was used in this study. Because of composition optimization and observation of its results, it was aimed to observe pH effects, and it was envisaged not to promote sucrose inversion through structural changes of autolyzed yeast cell proteins and oligosaccharides due to pH change. In addition, in previous studies, pH decreases (Romo-Zamarrón et al., 2019) or increases (Sumonsiri et al., 2021), depending on the properties of the substitute used, causing problems in terms of interpreting the findings and associating them with the alternative components used. As a result, the effect of pH on the product's primary characteristics can be neglected since it varies within a narrow range.

### 3.3 Color properties

In confectionery products, visual features are essential quality indicators associated with some sensory features and aroma release. Gummy candies, which have an opaque and yellowish appearance, are ideal products for using colorants. Natural pigments generally associated with the flavoring agents used are included in the composition for this purpose (Gunes et al., 2022). In this study, red beet extract, a source of betalain, was used as a colorant (Spórna-Kucab et al., 2013) to observe the effects of composition factors and storage conditions. The samples' L*, a*, b*, chroma, and hue angle values were determined as 61.5–86.8, 3.95–15.4, 1.93–13.2, 8.73–15.5, and 6.43–73.1, respectively. Although the samples are generally characterized by red color, the tone and intensity of this color varied over a wide range, and significant models were determined for all color parameters within the scope of the study (*P* < 0.05). The R^2^ values of these models are 0.6332–0.9606. In previous studies, the effects of various factors on gummy color characteristics have been determined. pH is the first variable to be considered for color properties. Because the colors and tones of various colorants and pigments are affected by the product's pH and matrix, the colorant selection must have the correct/targeted color properties as well as stability at the pH values of the products. Interactions between product ingredients and colorants should also be considered (Hubbermann, 2016). These interactions include the destabilization of anthocyanins with ascorbic acid (Sadilova et al., 2009) or interactions between pigments and flavoring substances (Dufour & Sauvaitre, 2000). In this study, the pH values of the samples were considered negligible due to their narrow range, which provided an advantage for examining the effects of independent variables on color properties. A study on gummies determined that heat treatment caused a decrease in L* value and an increase in a* and b* values (Wang & Hartel, 2022). In addition, factors affecting the rate of protein oxidation reactions may also cause changes in visual features (Bao & Ertbjerg, 2015). Another mechanism to consider is Maillard reactions. It has been emphasized that in model gel structures containing gelatin, where no colorant is used, the effects of this stabilizer concentration on color properties are less important compared to other process conditions (Dalabasmaz et al., 2023). Our findings demonstrated that gelatin concentration had a significant influence on the L* value, while the interactions between gelatin × sucrose and gelatin × autolyzed yeast cells also significantly affected L*, a*, chroma, and hue angle values. In contrast, the sucrose × gelatin interaction primarily influenced the b* value. These results indicate that the gel matrix composition directly alters light scattering and absorption properties, with higher gelatin concentrations increasing opacity and reducing translucency, thereby lowering L* values. The observed color variations with increased sucrose content can be attributed to non-enzymatic browning pathways, particularly Maillard reactions, which are promoted in the presence of amino groups from gelatin and reducing sugars, leading to the formation of brown pigments such as 5-HMF and furfural (Hubbermann, 2016). Furthermore, autolyzed yeast addition likely contributed to color changes through the release of low-molecular-weight amino acids and peptides, as well as degradation of yeast cell wall polysaccharides during autolysis and hydrolysis (Takalloo et al., 2020). The combination of these mechanisms suggests that yeast-derived components may act synergistically with gelatin and sucrose to influence pigment stability, particularly anthocyanin degradation, resulting in measurable differences in chromatic attributes. These findings emphasize the importance of controlling matrix composition when designing sucrose-reduced gummies to maintain color stability and consumer appeal.

### 3.4 Bioactive properties of gummy samples

The use of autolyzed yeast is gaining importance due to some of the bioactive components it contains. Autolyzed yeast cells are a rich source of functional biomolecules, particularly β-glucans, mannans, and bioactive peptides, which have been linked to various health-promoting properties (Bertolo et al., 2019; Liu et al., 2021). Additionally, low-molecular-weight compounds such as glutathione contribute to their antioxidant potential, while polyphenolic constituents further enhance their free radical scavenging capacity. In this study, enrichment of gummies with autolyzed yeast cells resulted in total polyphenol content (TPC) values ranging from 24.8 to 47.9 mg GAE/kg, representing a substantial improvement compared to conventional sucrose-based formulations. Statistical analysis revealed that increasing autolyzed yeast levels significantly elevated TPC values, and this relationship was well-described by a robust predictive model (R^2^ = 0.9749, *P* < 0.05). These findings align with previous reports demonstrating that yeast-derived cell wall components and associated metabolites contribute to improved antioxidant capacity in functional food matrices (Liu et al., 2021). Hence, the inclusion of autolyzed yeast not only reduces sucrose dependence but also provides an additional route for fortifying confectionery products with bioactive compounds, supporting their positioning as health-oriented alternatives. Previous studies emphasized that the yeast strain and the extraction method applied to the strain effectively affect the TPC value, which is 0.01–7.05 mg GAE/g (Datta et al., 2017). In our study, it was determined that the interaction between independent variables on the TPC values of gummy samples should be taken into account. Possible reasons for this situation include (i) the effects of sugar inversion and Maillard reaction products on polyphenols, (ii) the interaction of polyphenols and gelatin causes the formation of high molecular weight complexes, and the problems/limitations that may be experienced in their determination, (iii) the possible effects of composition changes in the gummy process on polyphenol stability. Increasing TPC using autolyzed yeast cells is essential in improving gummy candy's nutritional properties. Antioxidant activity potentials determined by the DPPH method also changed in line with the TPC results, and % inhibition values increased significantly with autolyzed yeast cells. It was determined that the amount of sugar and yeast cells significantly affected the antioxidant capacity (P < 0.05). This result is predictable because the amounts of these two components are interrelated in the study design. The determination of a significant model with a high R^2^ (0.9374) value for this dependent variable was among the remarkable results (P < 0.05).

### 3.5 *In vitro* bioactivity analysis

A two-way analysis of variance (ANOVA) was carried out to assess the effects of different nutritional treatments (gummy and yeast cell) and time on the viability of colon and lung carcinoma cells. There was a significant interaction between the independent and dependent variables (*p* < 0.0001), indicating that the effect of nutritional treatment on each cell viability depends on the time point. The decrease in cell viability over time varies across treatments, with the most substantial reduction observed in yeast treatments for both cell lines. Tukey's HSD post-hoc test was conducted to understand this interaction further and make subgroup comparisons. Gummy treatment for 24 h significantly reduced Caco-2 (55 %) and A549 (60 %) cell viability compared to the untreated control group (Fig. 2). The cytotoxic effects of these nutritional treatments were observed to be time-dependent, with a pronounced suppression in the viability of Caco-2 (∼35 %) and A549 cells (∼23 %) after 48 h of treatment in comparison to the untreated control cells (p < 0.0001). A dramatic reduction in cell survival rate (∼90 %) was observed in Caco-2 cells following exposure to yeast samples for 24 and 48 h (*p* < 0.001). Similarly, exposure to the yeast samples led to a marked decrease in cell survival rate (90 %) in A549 cells following 48 h treatment relative to untreated control cells (p < 0.001). “Furthermore, the inhibitory effect of yeast on cell survival was time-dependent, as cell viability in A549 cells decreased by 65 % relative to untreated controls following 24 h of treatment. Expectedly, DMSO treatment as a positive control exhibited a significant reduction (93 %) in cell survival in both cell lines.

### 3.6 FTIR spectra analysis

**Fig. 1:**
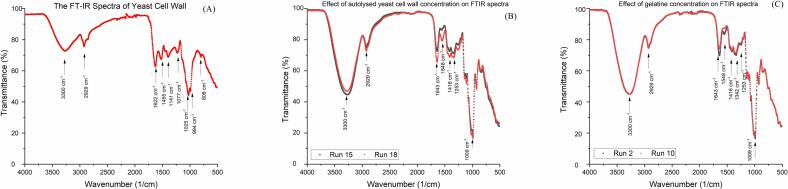
Fourier transform-infrared (FT-IR) spectra of gummy samples.

Fourier transform-infrared (FT-IR) spectroscopy monitors the structural changes in the samples. In Fig. 1, the autolyzed yeast cell structure was analyzed with FT-IR spectroscopy. The band observed around 994 cm^−1^ has been known as the β-1,6 Glucans, 1025 cm^−1^ belongs to β-1,4 Glucans, and 1077 and 1141 cm^−1^ bands belong to β-1,3 Glucans (Burattini et al., 2008; Galichet et al., 2001). FTIR analysis confirmed the presence of characteristic β-glucans in autolyzed yeast cells, as evidenced by the bands around 994 cm^−1^ (β-1,6-glucans), 1025 cm^−1^ (β-1,4-glucans), and 1077–1141 cm^−1^ (β-1,3-glucans), along with a distinct band near 808 cm^−1^ associated with mannans. The spectrum also exhibited peaks at 1455 cm^−1^ (CH₂/CH₃ bending of lipids and proteins) and 1622 cm^−1^ (amide I band, β-form C=O stretching of polypeptides), while absorptions at 2850 and 2930 cm^−1^ corresponded to symmetric and antisymmetric CH₂ stretching vibrations (Borchani et al., 2016). Comparative FTIR spectra of gummy formulations with varying yeast cell (Run 15–18) and gelatin concentrations (Run 2–10) revealed compositional effects on molecular interactions. The broad band around 3300 cm^−1^, associated with O—H stretching of bound water, decreased in intensity as autolyzed yeast concentration increased, indicating enhanced water-binding capacity imparted by yeast-derived polysaccharides and proteins. In contrast, gelatin concentration showed a negligible impact on this region, suggesting that water binding in these formulations is more strongly influenced by yeast-derived hydrophilic components than by gelatin network density. These findings demonstrate that autolyzed yeast acts as an active water-binding agent, potentially altering moisture distribution and textural stability in sucrose-reduced gummies, an effect that should be considered in formulation optimization.

**Fig. 2:**
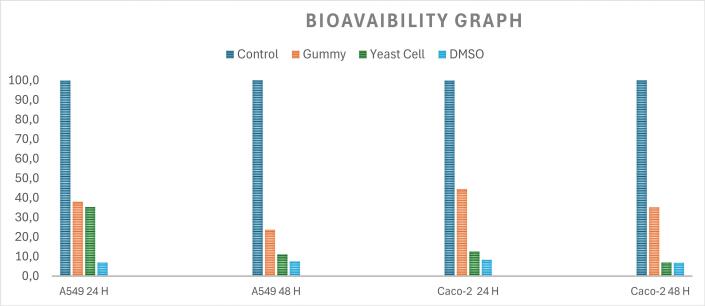
*In vitro* relative cell viabilities (Caco-2; Human colon carcinoma lines and lung (A54; lung carcinoma cell) of gummy samples.

**Fig. 3:**
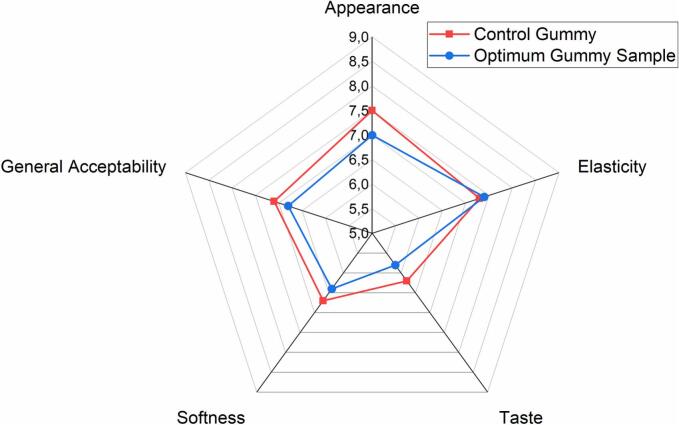
Gummy samples' sensory properties.

The bands with peak points around 2930 cm^−1^ were due to the contributions from C—H stretching vibrations of carboxylic acids and NH_3._ Increasing autolyzed yeast cell and gelatin concentrations caused a slight increase in peak intensity. The most significant band (Amide *I*) related to the gelatin is observed in the 1700–1600 cm^−1^ spectral range (Cebi et al., 2019). The peak around 1643 cm^−1^ increased significantly with gelatin and yeast cell concentrations. The spectral range between 1100 and 1000 cm^−1^ resulted from the (C—O) and (C-O-C) absorptions of the carbohydrates (Cebi et al., 2016). The peak (1009 cm^−1^) intensity did not change with gelatine concentration but increased slightly with autolyzed yeast addition. The peaks observed around 1255 cm^−1^ and 1415 cm^−1^ arise from the C—O stretching and O—H stretching/bending vibrations of carbohydrates, respectively (Tewari & Irudayaraj, 2004). This peak intensity increased with increasing autolyzed yeast cells and decreasing gelatine concentrations.

### 3.7 Sensory analysis

Sensory evaluation revealed that incorporation of autolyzed yeast cells improved the perceived elasticity of the gummy samples, which is consistent with the observed instrumental increase in springiness and resilience parameters (Fig. 3). However, slight reductions in appearance, softness, and taste scores were noted compared to the control formulation. These sensory changes may be attributed to the intrinsic color and flavor profile of yeast-derived components, as well as their influence on the microstructure of the gel matrix, which can modify mouthfeel and visual attributes. Similar findings have been reported when yeast extracts or cell wall components are integrated into bakery and confectionery products, where functional improvements were occasionally accompanied by flavor or color alterations requiring additional formulation adjustments (Pérez-Torrado & Querol, 2016). To mitigate these effects, targeted use of natural aroma agents or flavor maskers is recommended to balance the overall sensory profile while preserving the functional benefits of autolyzed yeast enrichment. This approach aligns with recent industry strategies to enhance nutritional quality without compromising consumer acceptability in functional confectionery products.

### 3.8 Accelerated shelf life

Confectionery products have a relatively longer shelf life than other foods. The high sugar content in these products makes them more resistant to microbiological spoilage. Therefore, physical and chemical changes such as aroma, texture, color, and flavor can be defined as factors and parameters that are more decisive on the shelf life of products. In this study, gummy samples containing different levels of sucrose, autolyzed yeast cells, and gelatin were stored under accelerated shelf-life conditions for 90 days. The samples' color and texture characteristics were determined at 7-day intervals. These parameters were chosen because (i) they are among the main quality attributes of gummies, (ii) the use of colorants and the use of colorants in commercial products, (iii) the strong relationship and correlation between gummy texture characteristics and other quality and shelf life parameters (such as moisture content, water activity). In general, the conformity assessment of a foodstuff is based on parameters such as color, texture, aroma, flavor, taste, shape, and characteristics that can be defined as abnormalities not associated with the product and microbial load. If a foodstuff deviates from the limit values set for these characteristics, it is considered spoiled and/or to have completed its shelf life (Nicoli & Calligaris, 2018).

In confectionery products, a ΔEa,b value of more than 3.0 is considered as the formation of a visible color difference (Periche et al., 2014). In the gummy samples, only one sample (8.00 g/100 g gelatin, 2.70 g/100 g autolyzed yeast cells and 29.90 g/100 g sucrose) had ΔEa,b < 3.0 at the end of the 90th day. This result can be stated as an adverse finding in general. However, it should be noted that the pH modification, which provides an advantage in terms of stability for the colorant, was not carried out using any organic acid. In addition, it was found remarkable that lower ΔEa,b values were determined for samples with relatively lower pH values. A significant model for the relationship between color change and dependent variables under accelerated shelf-life conditions was determined (*P* < 0.05). This result is promising for using the RSM approach for shelf life studies in confectionery products. Another finding was that the interaction of gelatin concentration with other independent variables significantly affected the increase in color stability (P < 0.05).

In the study, TPA analyses were performed periodically, and the different textural properties of the samples (hardness, springiness, cohesion, resilience, gumminess, chewiness) were determined. Among these properties, especially hardness is of critical importance. This parameter provides important information regarding determining the moisture migration that may occur in gummy candies. Determination of moisture content in the samples may cause some errors due to the presence of water that has migrated to the surface and may still be present in that area. However, the moisture-texture relationship is also critical and is often a limiting factor controlling shelf life during storage. Therefore, understanding moisture and its effects on confectionery is critical to maintaining quality and shelf life. These properties were found to have a strong relationship with water activity (Ergun et al., 2010). In this study, the change in hardness value varied over a wide range [(−1.39)-54.1 %], and a significant increase in hardness was observed. A substantial pattern for hardness change under accelerated shelf-life conditions was identified. In particular, interactions of gelatin concentration with other independent variables and sucrose-autolyzed yeast cell interactions were found to be important. This hardening is undesirable for product quality (Hartel et al., 2018).

As mentioned above, there is a relationship between the increase in hardness and the water activity values of the product. Because gummy candies tend to harden as they lose moisture to the environment. If gummies are stored in dry conditions due to aw values (between 0.5 and 0.7), they lose moisture and harden. However, in our study, the samples were stored in an environment with high relative humidity (70 % RH). Hardening was encountered due to the samples' relatively high water activity values. In addition, a high correlation was found between water activity values and hardness increase. In these systems, moisture control through hydrocolloid gelation is critical for product texture control (Ergun et al., 2010). However, in the study, the main effect of gelatin on hardness change was insignificant. The interactions of this component with other independent changes were significant (P < 0.05). This may be explained by the importance of the presence, types, and levels of saccharides in the medium in the formation and tightness of the gelatin network (Gok et al., 2020). Another possible reason is that the amounts of autolyzed yeast cells and sucrose had a more significant effect on the water activity values than the gelatin concentrations studied. It was also determined that the sucrose x autolyzed yeast interaction significantly affected the hardness change. The glass transition temperature (Tg) depends on the molecular weight, the degree of polymer crosslinking, and the plasticizer concentration (*e.g.*, water). Low molecular weight carbohydrates generally have lower Tg, while high molecular weight ones generally have higher Tg (Ergun et al., 2010). When different saccharides are mixed, as in most confectionery formulations, the Tg of the mixture depends on the relative proportions of the added saccharides, the Tg values of each sugar in the mixture, and the water content. As a result of the use of autolyzed yeast cells with sucrose substitution, the increase in the glass transition temperature due to the change in the average molecular weight of the saccharides in the composition affected the hardening tendency during storage. As mentioned above, there is a relationship between the hardness increase and the water activity values of the product. As a result, when autolyzed yeast cells are used to partially substitute sucrose and gelatin in gummy confectionery, reducing the water activity value would help prevent or limit the textural changes during the storage process. Ingredients that can be used for this purpose include organic acids, glycerol and sorbitol. Lowering the pH, especially by using organic acids, can also contribute to the increase of color stability and the reduction of ΔEa,b values in storage. However, the effects of these substances on sucrose inversion and autolyzed yeast cell oligosaccharides and proteins, as well as on the Maillard reaction and products that may occur during the process, will need to be studied.

### 3.9 Optimization study

Response surface methodology (RSM) optimization identified the optimal gummy formulation as 26.27 % sucrose, 6.33 % autolyzed yeast cells, and 7.00 % gelatin, simultaneously maximizing elasticity and bioactive properties while minimizing hardness and ΔE color changes during accelerated shelf-life storage. At this optimum, theoretical response predictions (Table 5) were validated experimentally, with deviations between predicted and observed values below 10 % for all parameters except springiness (14 %), which is still within the acceptable range reported for complex food matrix optimizations (Ravichandran et al., 2019). The slightly higher variation in springiness may be attributed to non-linear interactions between gelatin network density and the water-binding capacity of yeast-derived polysaccharides, which influence gel recovery properties under deformation. Importantly, this formulation supports partial sucrose replacement without compromising critical quality parameters, indicating that autolyzed yeast cells can be functionally integrated into gummy systems with minimal adjustments. Such integration not only improves antioxidant and bioactive properties but also meets consumer expectations for reduced-sugar, functional confectionery products, as highlighted by similar reformulation studies in gelatin-based matrices (Dalabasmaz et al., 2023; Ergun et al., 2010).

**Table 5:** Optimization of Process Conditions and Validation of Results.

**Optimum Points**	**Factor A**	**Factor B**	**Factor C**	**Response1**	**Response 2**	**Response 3**	**Response 4**	**Response 5**	
	Sucrose (g/100 g)	Dried Yeast Cell (g/100 g)	Gelatin (g/100 g)	Springiness (%)	Antioxidant Activity (Inh %)	TPC Content (mg GAE/kg)	ΔE	Hardness Variation (%)	Desirability
	26.27	6.33	7	49.78	15.47	54.24	3.35	12.08	0.717
**Experimental** **Validation**	**Factor A**	**Factor B**	**Factor C**	**Response 1**	**Response 2**	**Response 3**	**Response 4**	**Response 5**	
	Sucrose (g/100 g)	Dried Yeast Cell (g/100 g)	Gelatin (g/100 g)	Springiness (%)	Antioxidant Activity (Inh %)	TPC Content (mg GAE/kg)	ΔE	Hardness Variation (%)	
	26.27	6.33	7	42.63	14.69	49.36	3.12	11.23	
**Deviations (%)**				14	5.03	8.99	6.86	7.03	

## 4 Conclusion

This study demonstrated the technological feasibility and functional potential of partially replacing sucrose with spray-dried autolyzed *Saccharomyces cerevisiae* yeast cells in gummy formulations. The inclusion of yeast-derived β-glucans, mannans, peptides, and associated oligosaccharides altered gelatin network dynamics, enhanced water-binding capacity, and modified matrix interactions, resulting in distinct changes in textural attributes, particularly chewiness and springiness. Fourier-transform infrared (FTIR) spectroscopy confirmed structural modifications in protein–polysaccharide interactions and water association, evidencing the functional contribution of autolyzed yeast as both a bulking and bioactive ingredient.

Physicochemical analyses revealed that yeast enrichment increased water activity and affected moisture migration, leading to storage-associated hardening under high relative humidity conditions. Nevertheless, this challenge can be mitigated through compositional adjustments in hydrocolloids and incorporation of humectants or pH-modifying agents, which may also enhance color stability by reducing ΔE shifts during storage. Sensory analysis indicated improved elasticity but slight reductions in appearance, softness, and taste, suggesting that targeted flavor optimization is necessary to achieve full consumer acceptability.

From a nutritional perspective, autolyzed yeast inclusion substantially increased total phenolic content and antioxidant capacity while reducing overall sucrose content, meeting consumer and regulatory demands for lower-sugar, functionally enriched confectionery. The optimized formulation (26.27 % sucrose, 6.33 % autolyzed yeast cells, and 7 % gelatin) exhibited pronounced *in vitro* bioactivity, significantly suppressing Caco-2 and A549 carcinoma cell viability in a time-dependent manner, suggesting potential postbiotic-associated health benefits beyond conventional confectionery functionality.

Collectively, these findings highlight the potential of autolyzed yeast cells as a sustainable and multifunctional ingredient for developing next-generation, lower-sugar, postbiotic-enriched gummy confections. Future research should focus on optimizing flavor masking strategies, further improving moisture and color stability, and evaluating sensory acceptance and *in vivo* functional effects to accelerate industrial application and commercialization of such bioactive confectionery products.

## CRediT authorship contribution statement

**Tahra ElObeid:** Writing – review & editing. **Burcu Tüzün:** Formal analysis. **Ayşe Apaydın:** Formal analysis. **Omer Said Toker:** Writing – review & editing. **Caglar Doguer:** Formal analysis. **Ibrahim Palabiyik:** Writing – review & editing, Writing – original draft. **Muhammed Irfan Aksu:** Writing – review & editing, Formal analysis. **Nevzat Konar:** Writing – review & editing. **İlyas Atalar:** Writing – review & editing, Supervision, Methodology, Conceptualization.

## Declaration of competing interest

The authors declare that they have no known competing financial interests or personal relationships that could have appeared to influence the work reported in this paper.

## Data Availability

The data will be available from the corresponding author.
